# The COVID-19 pandemic: critical issues and perspectives for infectious disease prevention in Africa

**DOI:** 10.1080/20008686.2020.1798073

**Published:** 2020-07-31

**Authors:** Ayodele Oluwaseun Ajayi

**Affiliations:** Department of Microbiology, Federal University Oye Ekiti, Oye Ekiti, Nigeria ayodele.ajayi@fuoye.edu.ng

## Introduction

The current COVID-19 caused by the SARS-CoV-2 is presently rattling global public health with huge numbers of fatalities and vast geographical spread, compared with other recent pandemics like the severe acute respiratory syndrome (SARS) and the Middle East respiratory syndrome. The pandemic reportedly originated from a wildlife animal market in China, although there are differing reports regarding the actual origin in China of the virus directly responsible for this disease [1].

Compared with other pathogens that were implicated in recent pandemics, the SARS-CoV-2 shows distinction in its rapid spread and virulence. The virus is transmitted through the respiratory tract by direct close contact with infected people and can be shed asymptomatically, thereby increasing its chance to cause large-scale infections among humans [2,3]. Contaminated surfaces or objects can also serve as vehicles of transmission, increasing the risk of infection among individuals. Although the details of the virulence mechanisms of this virus are still emerging, the scale of infections globally and the fatality it causes can be attributed to its high transmissibility. According to the Johns Hopkins Coronavirus Resource Center [4], the total confirmed cases of infection currently stand at more than 11.6 million with more than 540,000 deaths globally. Although the Americas and Europe appear to be the worst hit, and with Latin America emerging as the new epicentre of the disease, the growing morbidity and mortality of the disease in the African countries are a subject for a serious public health concern, with implications for public health and considerations for prevention of such infectious diseases in future.

In Africa, the disease started with an index case in Algeria and has spread to more than 45 countries. According to the WHO’s situation update on COVID-19 for Africa, the number of cases is more than 304,000 and more than 6155 deaths as on 1 July 2020 [5]. Although these numbers of reported cases are lower compared to the morbidity and mortality in the rest of the world, the accelerated spread of the virus in developed countries with more developed healthcare systems should bother public health policy specialists and authorities in Africa. This is because of the less developed public healthcare systems in many African countries, easily becoming overwhelmed with a rapid increase in cases. In view of the global impact of COVID-19, this opinion, therefore, seeks to highlight the critical aspects that need to be strengthened to prevent and control the emergence of such diseases in future. The critical aspects already identified are as follows:

### Surveillance

The COVID-19 pandemic has shown that surveillance must be prioritized in order to forestall future pandemics in Africa. The major aim of such surveillance efforts should be to identify possible clusters and origins of infectious pathogens and the population at risk. Two major aspects need to be prioritized in future surveillance efforts in Africa. The first is highly trained epidemiologists and public health workforce for surveillance and the second is the technology. Additional personnel should be trained and deployed strictly for surveillance to detect possible infectious pathogens. This should be in addition to the existing workforce for surveillance and epidemiological investigations during disease outbreaks. In terms of technology, pathogenomics and whole-genome sequencing are gradually becoming gold standards for disease surveillance [6]. The technology and expertise for such genomic surveillance efforts are grossly lacking in Africa. These core aspects need to be developed and deployed immediately to scale up the ability to predict the emergence of infectious diseases in future. Microbiology and public health laboratories in Africa should be assisted to acquire gene and genomic sequencers and data infrastructure for surveillance of emerging infectious disease pathogens. Mathematical modelling techniques can be incorporated to increase the predictive power and accuracy of such surveillance.

### Biotechnology

Some countries like South Korea, Germany and Taiwan responded promptly and effectively controlled the pandemic. They relied on their biotechnological expertise, and this should serve as an example for Africa. These countries promptly deployed necessary expertise within a short period to massively produce test kits immediately the first cases were confirmed. Millions of tests were carried out to identify clusters of the disease and asymptomatic carriers of the virus quarantined [7]. The biotechnological capacity, expertise and infrastructure in Africa need to be improved significantly to enhance the response of individual countries to any future emergence of infectious diseases. African countries should be assisted with funding and infrastructure for research and development to enhance their biotechnological capacity. Biotechnology-based industries can produce diagnostic test kits and vaccines to detect infected individuals and prevent spread within the population. Majority of such products are imported from other countries, and this severely hinders mitigatory efforts by public health specialists and laboratory personnel to detect and curb the spread of infectious agents.

### One Health

The first details about the emergence of the COVID-19 indicated that the pathogen emerged from a wildlife animal market in Wuhan, China, and subsequently spread to more than 90 countries across the globe. This implies that certain aspects of this disease can be managed with One Health, which is a multidisciplinary approach that emphasizes the increasing interconnectedness between humans, animals and environmental health. The increasing urbanization in most African countries and cities ensures that human–animal ecosystems are increasingly becoming less distinct. This increases human–animal contacts with increasing chances of transfer and exchange of pathogenic microorganisms. Africa currently has a substantial public health burden in terms of morbidity and mortality due to other zoonotic viral infections like Ebola fever, yellow fever, rabies and Lassa fever. This buttresses the importance of One Health as an aspect of pathogen and infectious disease surveillance. Fortunately, some countries in Africa have seen the importance of this concept and have prioritized various policies to promote One Health as a component of their cardinal public health programs [8,9]. An urgent need at the moment is for African countries to intensify this approach and create a more multidisciplinary expertise that will cut across human, animal and environmental health to better understand the complexities between these components with an emphasis on the types of pathogens that can emerge and how to prevent their transmission in the future.

### Burden of other infectious diseases

The academic and scientific communities responded promptly to the COVID-19 pandemic with more attention and funding to address the concerns of the pandemic to better understand the aetiology, epidemiology and prevention [10]. In view of the scale of this pandemic, it is likely that more attention and funds will be committed to fight the disease in years to come, with less emphasis on the infectious diseases that are currently ravaging the African continent before the COVID-19 pandemic. Although the number of cases in Africa is still low, other viral infectious diseases are still plaguing the continent. About 11,000 fatalities were recorded during the last Ebola virus disease outbreak that occurred in Sierra Leone, Liberia and Guinea in 2014–2016 [11]. The Democratic Republic of Congo recorded more than 2000 fatalities in the latest Ebola virus disease outbreak in the country. Nigeria is still in the midst of a Lassa fever outbreak that has claimed more than 100 lives shortly before the explosion of the COVID-19 pandemic. Malaria is endemic in the African continent, and it continues to claim the lives of thousands of children. Tuberculosis still poses a serious health burden in Africa [12]. Diarrheal diseases still remain a huge public health and economic burden in the African continent [13]. In 2012, infantile pneumonia, together with malaria and diarrhoea, accounted for more than 2 million deaths in children less than 5 years in Africa. In the post-COVID-19 period, the priority attention given to these infections stands at a risk of diminishing in Africa [14]. Any potential gain from systematic efforts to fight COVID-19 could diminish if attention is significantly shifted away from other infectious diseases common in Africa. In conclusion, African governments need to increase budgetary attention to healthcare, research and development in order to prevent costly surges in these infections and with a view to improving overall public health.
